# Combined neoadjuvant chemotherapy and immunotherapy in a hepatitis B virus-positive patient with locally advanced rectal adenocarcinoma: a case report and literature review

**DOI:** 10.3389/fonc.2025.1560508

**Published:** 2025-08-20

**Authors:** Qiyun Li, Xue Wu, Zhibin Xu, Taidong Li

**Affiliations:** ^1^ Department of Medical Oncology, Central Hospital of Guangdong Provincial Nongken, Zhanjiang, Guangdong, China; ^2^ Department of Organ Transplantation, The First Affiliated Hospital of Guangzhou Medical University, Guangzhou, Guangdong, China; ^3^ Department of Surgical Oncology, Central Hospital of Guangdong Provincial Nongken, Zhanjiang, Guangdong, China

**Keywords:** neoadjuvant therapy, immune checkpoint inhibitors, colorectal cancer, locally advanced rectal cancer, cmFOLFOXIRI

## Abstract

Microsatellite-stable (MSS) rectal adenocarcinoma remains a therapeutic challenge, particularly in patients with complicating factors such as chronic hepatitis B virus (HBV) infection. Advances in immunotherapy, including immune checkpoint inhibitors (ICIs), have introduced new opportunities to improve the treatment outcomes in this subset, yet their application in HBV-positive cancer patients is less well understood. Here we report the case of a 46-year-old female with MSS locally advanced rectal adenocarcinoma and active HBV infection, successfully treated with cmFOLFOXIRI combined with camrelizumab as neoadjuvant therapy. The patient presented with a circumferential rectal mass, elevated tumor markers, and virological evidence of high HBV viral load, necessitating prophylactic antiviral management with entecavir. Following five cycles of cmFOLFOXIRI and two cycles of camrelizumab, significant tumor regression was achieved, with further response observed after long-course radiotherapy combined with irinotecan and capecitabine. Laparoscopic low anterior resection revealed complete pathological remission (pCR), with no residual tumor cells or lymph node metastases identified. This case underscores the potential of integrating immunotherapy into multimodal neoadjuvant regimens for MSS rectal cancer while highlighting the critical importance of HBV management to minimize reactivation risks during treatment. These findings offer valuable insights into the safe and effective use of ICIs in HBV-positive cancer patients, warranting further investigation in larger clinical studies.

## 1 Introduction

Colorectal cancer (CRC) is a malignancy associated with both high incidence and mortality worldwide. According to World Health Organization (WHO), an estimated 1.9 million new CRC cases were diagnosed globally in 2020, accounting for approximately 9.7% of all newly reported malignancies (1). CRC currently ranks as the third most commonly diagnosed cancer, following lung and breast cancers. Although historically more prevalent in developed countries, the incidence of CRC has also been rising in developing regions. In China, for example, more than 270,000 new cases and approximately 130,000 deaths were reported in 2020; notably, there is an increasing trend of onset at younger ages (2, 3).

Locally advanced rectal cancer, defined as a tumor extending to adjacent organs or the peritoneum (T4) without distant metastases, poses a substantial therapeutic challenge due to high recurrence rates (4). Although surgery remains the cornerstone of treatment, the complexity and extent of tumor infiltration often limit the likelihood of complete resection and increase the risk of disease recurrence, which has been reported in approximately 25% to 40% of cases (5). For patients initially deemed unsuitable for curative surgery, neoadjuvant chemoradiotherapy is the standard approach. By reducing tumor burden preoperatively, this strategy improves resectability and lowers postoperative recurrence rates (6). However, conventional neoadjuvant chemoradiotherapy alone may not fully address the needs of patients with inherently low tumor immunogenicity, limiting the overall treatment efficacy in this subset.

The recent years have witnessed the emergence of immunotherapy—particularly immune checkpoint inhibitors targeting PD-1—as a promising therapeutic avenue for CRC. In the CheckMate 8HW trial (7), first-line nivolumab (a PD-1 inhibitor) combined with ipilimumab (a CTLA-4 inhibitor) in patients with MSI-H/dMMR metastatic CRC yielded a 2-year progression-free survival (PFS) rate of 72%, compared with 14% for chemotherapy. Combination regimens that incorporate immunotherapy are now an active area of research, aimed at optimizing the tumor microenvironment, enhancing immune responses and improving clinical outcomes. Nonetheless, for patients whose tumors exhibit low immunogenicity (e.g., pMMR/non–MSI-H) or those with locally advanced disease, conventional neoadjuvant chemotherapy remains integral to improving surgical resectability and overall treatment success.

Among neoadjuvant chemotherapy options, the FOLFOXIRI regimen (5-FU/leucovorin, oxaliplatin, and irinotecan) has demonstrated superior objective response rates (ORR), PFS, and overall survival (OS) compared with traditional doublet regimens such as FOLFOX (oxaliplatin plus 5-FU/leucovorin) or FOLFIRI (irinotecan plus 5-FU/leucovorin) in advanced CRC (8). As a result, authoritative guidelines issued by the National Comprehensive Cancer Network (NCCN) (9), the European Society for Medical Oncology (ESMO) (10), and the Chinese Society of Clinical Oncology (CSCO) (11) endorse FOLFOXIRI as a standard treatment option for advanced disease.

Despite its demonstrated efficacy, the toxicity profile of FOLFOXIRI, particularly in Asian populations, can limit its clinical application. To address these tolerability concerns, the Chinese modified FOLFOXIRI (cmFOLFOXIRI) regimen was developed. By adjusting doses and administration schedules—such as using 85 mg/m^2^ of oxaliplatin and 150–165 mg/m^2^ of irinotecan, along with optimized doses of 5-FU and leucovorin—cmFOLFOXIRI improves tolerability and reduces adverse events, thus increasing its feasibility for Asian patients (3).

Hepatitis B virus (HBV) infection, prevalent in China and Southeast Asia, adds another layer of complexity to CRC management. Chronic HBV infection can induce immunosuppressive states that may influence the efficacy of both chemotherapy and immunotherapy. HBV carriers often experience prolonged immune tolerance, which may alter their response to oncologic treatments compared to non-infected individuals. Further clinical investigation is needed to define safe and effective therapeutic strategies for this unique patient population (12).

While cmFOLFOXIRI has gained acceptance as a first-line therapy for advanced CRC, its role in the treatment of locally advanced disease remains less well established. Through a case report and literature review, we present a patient with locally advanced rectal adenocarcinoma who was an HBV carrier and achieved complete response following a combined treatment with cmFOLFOXIRI and immunotherapy. Our findings provide preliminary insights that may inform future research and guide clinical decision-making for this emerging therapeutic approach in locally advanced CRC.

## 2 Case presentation

### 2.1 Patient information

The patient is a 46-year-old female, married, with no history of smoking, excessive alcohol consumption, or major hereditary diseases. She has a history of chronic hepatitis B virus (HBV) carrier status and reported the detection of HBV infection in 2020 but did not receive standardized treatment. Hospital admission revealed a positive hepatitis B e antigen (HBeAg) with high viral load. In early 2023, she presented with a 1-month history of intermittent hematochezia and diarrhea but reported no notable abdominal pain, weight loss, or other systemic symptoms. Upon examination, the patient appeared to be in good general health. Her Eastern Cooperative Oncology Group (ECOG) performance status was 1, indicating that she was ambulatory and able to carry out light work.

No abnormal superficial lymph nodes were palpable. The abdomen was flat, without engorged abdominal wall veins or visible peristaltic waves. Palpation revealed a soft, nontender abdomen with no rebound tenderness or detectable masses. The liver and spleen were not palpable, and there was no tenderness along the ureteral pathway. Murphy’s sign was negative. There was no costovertebral angle tenderness, no shifting dullness, and normal bowel sounds were observed.

### 2.2 Clinical presentation and auxiliary examinations

The patient was admitted with a history of intermittent hematochezia and diarrhea persisting for over 2 years. Digital rectal examination revealed a complex, poorly demarcated, and fixed lesion located approximately 5 cm from the anal verge. Colonoscopy further characterized the lesion as a circumferential rectal mass extending from the mid to lower rectum (5 to 10 cm from the anal verge) with an irregular surface, marked congestion, and bleeding, resulting in significant luminal narrowing (**Supplementary Figure S1**). The biopsy pathology revealed a well-differentiated adenocarcinoma, and immunohistochemical analyses (**Supplementary Figures S2A, B**) demonstrated positive staining for CK20 and SATB2, a 90% Ki-67 labeling index, p53 positivity in a mutant pattern (≥70% of cells), and CEA positivity, all consistent with an adenocarcinoma phenotype. Mismatch repair (MMR) testing revealed a proficient MMR (pMMR) status. Imaging studies further delineated local extension and potential metastatic spread. Endoscopic ultrasonography, performed approximately 1 week after admission, demonstrated that the rectal tumor involved the entire bowel wall thickness and reached the serosal layer yet retained a clear boundary with the uterus and vaginal wall. Multiple hypoechoic lymph nodes were identified adjacent to the rectum, the largest measuring 8.1 mm, suggesting possible nodal metastases (**Supplementary Figures S3A, B**). The pelvic CT scans showed mural thickening of the mid-rectum and multiple enlarged perirectal lymph nodes (>8 mm in short-axis diameter) (**Supplementary Figures S4A–C**). The chest and abdominal CT imaging, conducted shortly after admission, revealed mild chronic inflammation in the right lower lobe of the lung, signs of cirrhosis, and slightly enlarged retroperitoneal lymph nodes but no definitive evidence of distant metastases (**Supplementary Figures S5A, B**). The whole-body bone scintigraphy (ECT) showed no bone metastases (**Supplementary Figure S6**). The laboratory tests revealed elevated tumor markers: CEA at 46.91 ng/mL, CA19–9 at 56.29 ng/mL, and CA242 at 18.16 ng/mL, indicating high metabolic activity of the malignancy and supporting the likelihood of a malignant process. As an HBV carrier, the patient’s virologic profile showed HBsAg >250 IU/mL, HBeAg 15.8 IU/mL, and positive HBsAb and HBcAb, with a low HBV-DNA viral load (<1.00 × 10² IU/mL). The liver function tests indicated mildly elevated aspartate aminotransferase (AST, 41.2 U/L) and gamma-glutamyl transferase (GGT, 72.0 U/L), while the other parameters were within normal limits.

### 2.3 Diagnostic assessment

Integrating the patient’s clinical history, imaging findings, and pathologic results, the preliminary diagnosis was mid-rectal adenocarcinoma (cT4aN1bM0, pMMR, stage IIIB) (AJCC 8th), with the tumor extending into the rectal serosa and likely involving perirectal lymph nodes. Molecular profiling of the tumor revealed microsatellite stability (MSS), a KRAS G12V mutation, MET gene amplification, and CCND1 gene amplification, indicating an aggressive molecular phenotype with potential implications for targeted therapy. Given the patient’s active hepatitis B status (HBsAg >250 IU/mL, HBeAg positive), there was concern about potential viral reactivation during immunotherapy and chemotherapy. Following a multidisciplinary team (MDT) discussion, an individualized treatment approach was outlined: initiate neoadjuvant chemotherapy with cmFOLFOXIRI combined with camrelizumab and then evaluate the extent of tumor regression to determine the subsequent use of radiotherapy and, potentially, surgical intervention. Rectal protocol MRI was not performed at any point during the diagnostic or treatment process due to patient-specific contraindications (e.g., claustrophobia or incompatible implants), leading to reliance on pelvic CT and endoscopic ultrasonography for staging and follow-up assessments.

### 2.4 Treatment course and therapeutic outcome assessment

**Figure 1:**
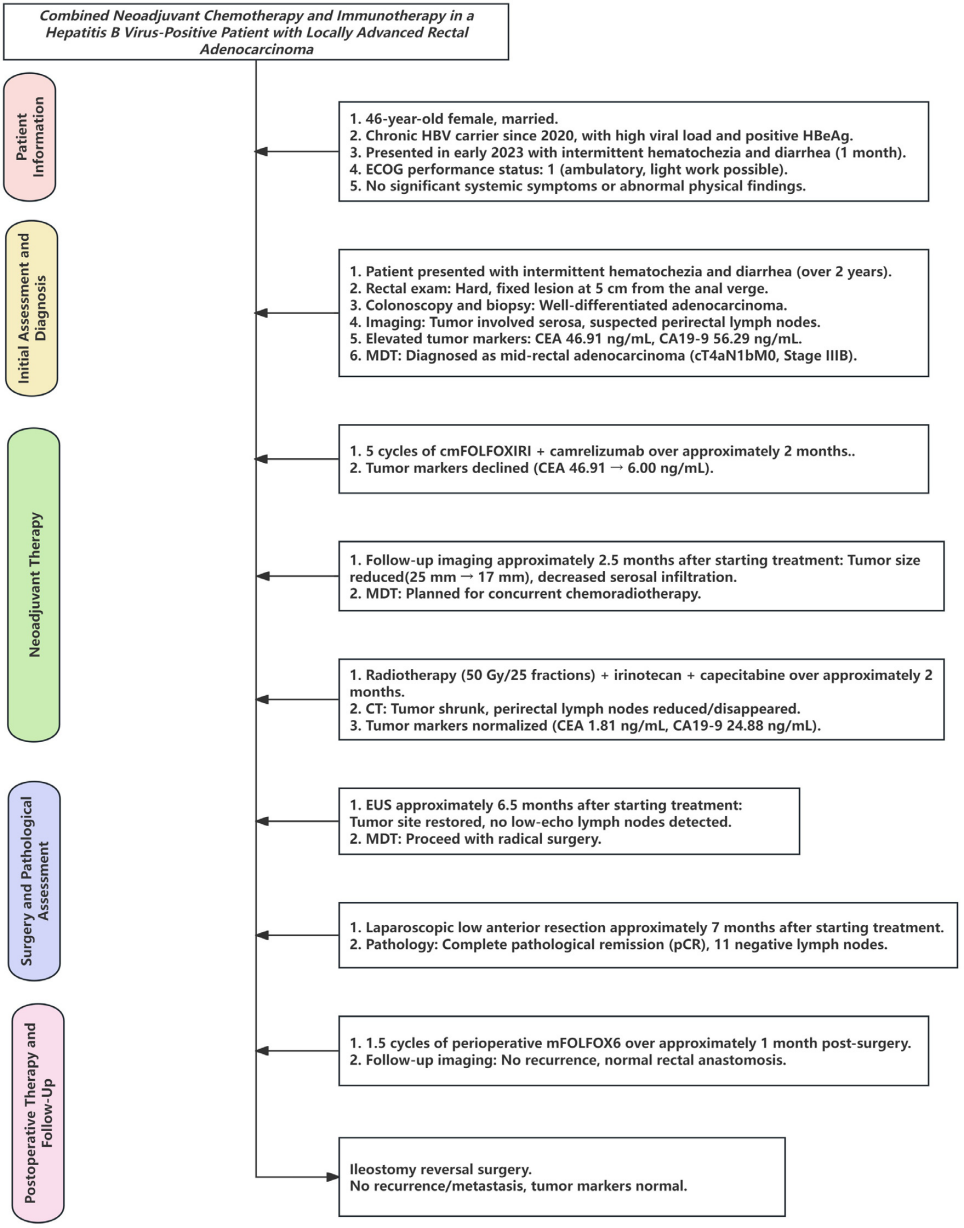
Diagnosis and treatment flowchart.

At initial presentation, the patient presented with a 2-year history of intermittent hematochezia and diarrhea. The digital rectal examination revealed a rigid, fixed, circumferential lesion located 5 cm from the anal verge. Colonoscopy identified a circumferential mass extending from the mid to lower rectum with an irregular surface, marked congestion, and bleeding. The pathologic evaluation of biopsy specimens confirmed a diagnosis of well-differentiated adenocarcinoma. Abdominal CT imaging demonstrated irregular thickening of the left rectal wall in the mid-rectum, involving the serosal layer, and multiple enlarged perirectal lymph nodes (maximum diameter, 8 mm × 7 mm), suggesting possible lymph node metastases. The laboratory investigations revealed significantly elevated tumor markers, including AFP at 14.21 ng/mL, CEA at 46.91 ng/mL, and CA19–9 at 56.29 ng/mL (all above the reference ranges). Hepatitis B virus (HBV) serology revealed HBsAg levels exceeding 250 IU/mL and HBeAg levels of 15.8 IU/mL (both elevated), with an undetectable HBV-DNA viral load. The liver function tests showed mildly elevated AST (41.2 U/L) and γ-GGT (72.0 U/L). Antiviral therapy with entecavir (Huadong Medicine Co., Ltd.) was initiated to address the patient’s chronic HBV infection (**Figure 1**).

To prevent HBV reactivation during multimodal therapy, entecavir prophylaxis was maintained throughout treatment. During neoadjuvant chemotherapy (over approximately 2 months, cmFOLFOXIRI plus camrelizumab, 5 cycles), HBV-DNA remained undetectable (<1.00 × 10² IU/mL, tested biweekly), with ALT fluctuating between 19.0 and 61.0 U/L (peak: 61.0 U/L), AST 17.0–61.8 U/L (peak: 61.8 U/L), and γ-GGT 42.0–80.0 U/L (peak: 80.0 U/L). These transient elevations, likely due to chemotherapy-induced hepatotoxicity or underlying cirrhosis, resolved without clinical hepatitis. During the neoadjuvant therapy with cmFOLFOXIRI and camrelizumab, the patient experienced mild fatigue, characterized by temporary tiredness that occurred intermittently during the treatment cycles. This adverse reaction was manageable with adequate rest and did not necessitate modification of treatment or discontinuation of therapy. The fatigue resolved spontaneously between cycles, consistent with the known safety profiles of the administered agents.

At approximately 2 weeks after initial presentation, the patient received 5 cycles of neoadjuvant chemotherapy with the cmFOLFOXIRI regimen combined with camrelizumab over 2 months. The chemotherapy regimen included irinotecan (150 mg/m^2^, Chia Tai Tianqing Pharmaceutical Group Co., Ltd.), oxaliplatin (85 mg/m^2^, Jiangsu Hengrui Pharmaceuticals Co., Ltd.), and leucovorin (400 mg/m^2^, Jiangsu Hengrui Pharmaceuticals Co., Ltd. or Henan Furen Huaiqing Tang Pharmaceutical Co., Ltd.) via intravenous infusion, and 5-fluorouracil (5-FU, 2,400 mg/m^2^, Hainan Zhongtai Pharmaceutical Co., Ltd.) was administered via a 46-h continuous infusion every 2 weeks (q2w). Immunotherapy consisted of camrelizumab (200 mg per dose, Jiangsu Hengrui Pharmaceuticals Co., Ltd.) administered intravenously every 4 weeks for 2 cycles. During this period, the tumor markers declined progressively, with CEA decreasing from 46.91 to 6.00 ng/mL and CA19–9 from 56.29 to 66.11 ng/mL (still above the reference range).

Approximately 2.5 months after initiating neoadjuvant chemotherapy, a follow-up CT scan showed a reduction in the size of the circumferential rectal tumor on the left rectal wall, with a decrease in wall thickness from approximately 25 to 17 mm. The tumor still involved the serosal layer (**Supplementary Figure S7A**). The serosal infiltration appeared reduced, and the surrounding fat planes became more distinct, with no significant changes in perirectal lymph nodes (**Supplementary Figure S7B**). The digital rectal examination at this point revealed softening of the previously complex lesion, with reduced fixation and decreased size, though still palpable approximately 5 cm from the anal verge. An endoscopic evaluation (colonoscopy) revealed partial regression of the circumferential mass, with a less irregular surface, reduced congestion and bleeding, and improved luminal patency. A multidisciplinary team (MDT) discussion recommended neoadjuvant concurrent chemoradiotherapy.

**Figure 2:**
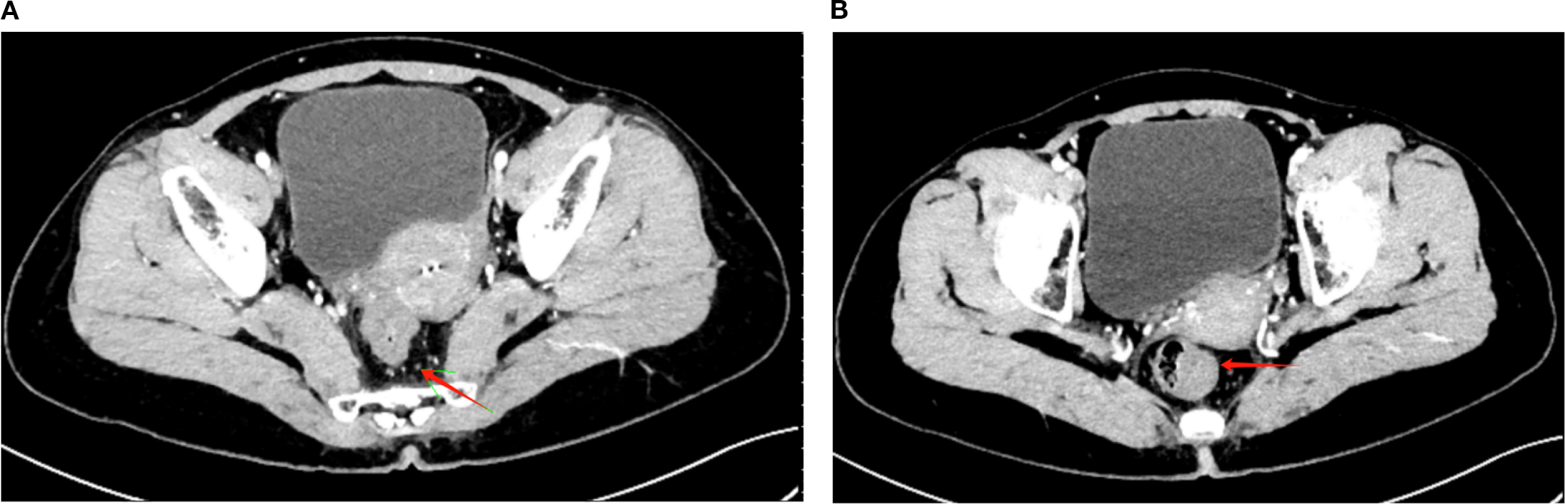
Post-treatment CT imaging showing tumor regression and lymph node reduction. **(A)** Axial CT image demonstrating a significant reduction in the rectal tumor volume (red arrow) following long-course radiotherapy (50 Gy/25 fractions) combined with concurrent chemotherapy (irinotecan + capecitabine). The serosal involvement is notably reduced, with clearer surrounding fat planes. **(B)** Axial CT image showing the reduced size of perirectal lymph nodes (red arrow), with partial regression of some nodes and no newly enlarged or suspicious lymph nodes observed.

Starting approximately 3 months after the initial chemotherapy, the patient underwent a long-course radiotherapy (50 Gy over 25 fractions) combined with concurrent chemotherapy over 2 months. The chemotherapy regimen included irinotecan (65 mg/m^2^, Chia Tai Tianqing Pharmaceutical Group Co., Ltd.) and capecitabine (625 mg/m^2^, Qilu Pharmaceutical Co., Ltd.). During chemoradiotherapy (February–March 2024), HBV-DNA remained undetectable (<1.00 × 10^2^ IU/mL, tested monthly), with ALT levels ranging from 18.0 to 29.0 U/L, AST levels ranging from 16.5 to 26.8 U/L, and γ-GGT levels ranging from 36.0 to 57.0 U/L, indicating stable liver function with no evidence of HBV reactivation. After chemoradiotherapy, CT revealed further tumor shrinkage, with a marked reduction in the area of serosal involvement and restoration of clear fat planes. The perirectal lymph nodes were reduced in size, with some completely regressing (**Figures 2A, B**). The tumor markers normalized, with CEA declining to 1.81 ng/mL and CA19–9 to 24.88 ng/mL. The digital rectal examination demonstrated further softening and near-resolution of the palpable mass, with minimal fixation. An endoscopic evaluation revealed significant tumor regression, with the mass appearing flatter, less congested, and without active bleeding, thereby allowing for better visualization of the lumen.

Immediately following chemoradiotherapy, the patient received 2 cycles of interval chemotherapy with the mFOLFOX6 regimen over 1 month. The regimen consisted of oxaliplatin (85 mg/m^2^, Jiangsu Hengrui Pharmaceuticals Co., Ltd.) and leucovorin (400 mg/m^2^, Henan Furen Huaiqing Tang Pharmaceutical Co., Ltd.) via intravenous infusion and 5-FU (400 mg/m^2^ as a bolus, followed by 2,400 mg/m^2^ via a 46-h continuous infusion, Hainan Zhongtai Pharmaceutical Co., Ltd.) every 2 weeks (q2w). In contrast to the previous cmFOLFOXIRI regimen, the transition to mFOLFOX6 was implemented to mitigate the risk of bone marrow suppression following concurrent chemoradiotherapy, thereby ensuring the completion of adjuvant chemotherapy. During this period, the tumor markers remained near normal, with CEA at 3.05 ng/mL (within normal range) and CA19–9 at 38.15 ng/mL (mildly elevated).

**Figure 3:**
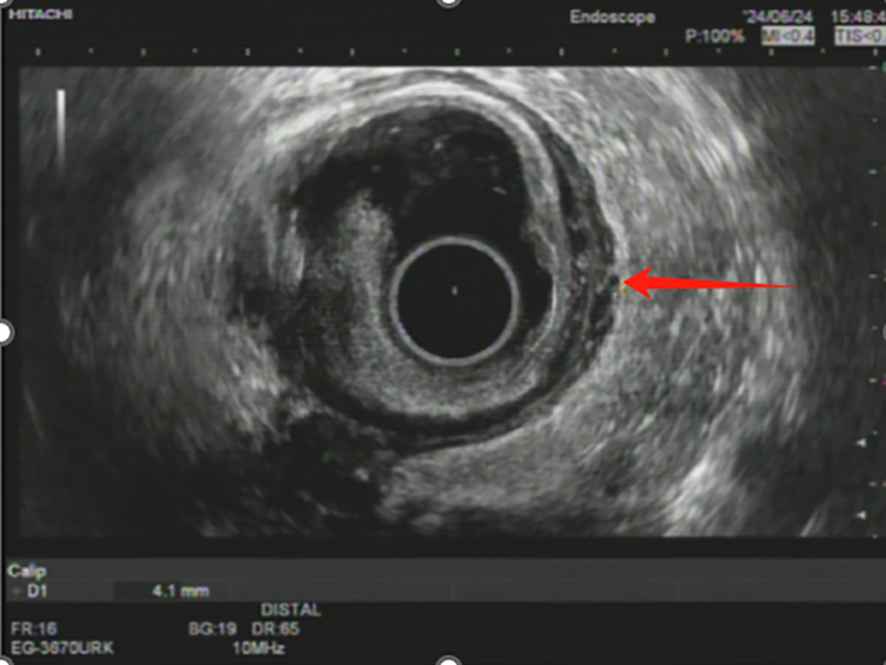
Endoscopic ultrasonography image showing post-treatment changes in rectal wall. The endoscopic ultrasonography (EUS) image demonstrates restoration of the rectal mucosa (red arrow) following multimodal therapy. The muscularis propria shows a layered structure with slight thickening (4.1 mm), consistent with treatment response.

Approximately 6.5 months after initiating neoadjuvant therapy, an endoscopic ultrasonography revealed the rectal lesion located 8–10 cm from the anal verge. The mucosa appeared restored at the tumor site, while the muscularis mucosa and submucosa exhibited hypoechoic changes and slight heterogeneity, with a thickness of approximately 1.6 mm. The muscularis propria had regained its layered structure but was slightly thickened at 4.1 mm. No low-echo lymph nodes were detected in the perirectal area, and clear anatomical separation was observed between the rectum and adjacent structures, including the bladder, uterus, and vaginal wall (**Figure 3**). Prior to EUS, digital rectal examination showed no palpable mass, suggesting complete clinical resolution at the rectal site. Endoscopic evaluation confirmed the restoration of normal mucosal appearance, with no visible residual tumor, irregular surface, or luminal narrowing. A third MDT discussion concluded that the patient was suitable for radical surgery.

Approximately 7 months after initiating neoadjuvant therapy, the patient underwent successful laparoscopic low anterior resection with regional lymphadenectomy and double-lumen ileostomy under general anesthesia. A pathological examination revealed no residual tumor cells in the rectal tumor bed after extensive sampling, with findings limited to clusters of histiocytes and fibrous stromal hyperplasia, consistent with post-treatment changes. A microscopic evaluation confirmed a complete pathological response (pCR), characterized by the total absence of viable tumor cells across all sampled sections of the rectal tumor bed. The residual tissue exhibited extensive fibrous stromal hyperplasia with scattered inflammatory infiltrates, predominantly composed of histiocytes and lymphocytes, indicative of treatment-induced remodeling. No evidence of residual adenocarcinoma or atypical cells was identified. An examination of 11 lymph nodes (six intermediate, two central, and three pericolic) showed no metastatic involvement, with the lymph nodes displaying standard architecture or mild reactive changes, further supporting the absence of residual disease.

The pathological response was graded as 0 (complete response), with no residual tumor cells observed microscopically. The proximal and distal resection margins were tumor-free. An immunohistochemical analysis showed CK-P negativity and CD68 positivity (indicative of histiocytes), further supporting a complete pathological response (pCR). During perioperative mFOLFOX6 chemotherapy, HBV-DNA increased slightly to 387 IU/mL but remained below reactivation thresholds, with ALT 13.0–41.0 U/L, AST 15.0–47.0 U/L, and γ-GGT 21.0–35.0 U/L. These mild fluctuations were not clinically significant, and entecavir prophylaxis continued without adherence issues or dose adjustments. Overall, the pathological results confirmed a complete pathological remission following multimodal therapy, with no residual tumor at the resection margins or in the lymph nodes (**Supplementary Figures S8A, B**).

Starting at approximately 1 month after surgery, the patient received 5 cycles of perioperative chemotherapy with the mFOLFOX6 regimen over 2 months. The treatment was well tolerated, with no significant adverse effects reported. At approximately 1.5 months after surgery, follow-up imaging revealed a normal appearance of the rectal anastomosis with no evidence of recurrence. The tumor markers, including CEA and CA19-9, remained within normal limits.

### 2.5 Surveillance

At approximately 4 months after surgery, the patient successfully underwent ileostomy reversal surgery, with an uneventful postoperative course and no significant complications.

At follow-up approximately 16 months after the initial presentation, the chest and abdominal CT scans revealed no evidence of recurrence or metastasis. The rectal anastomosis site was intact with a normal structural appearance, and the surrounding fat planes were well defined. The tumor markers were within normal limits, with AFP at 4.04 ng/mL, CEA at 3.28 ng/mL, and CA19–9 at 26.56 U/mL. Hepatitis B-related tests indicated HBsAg levels greater than 250 IU/mL, with a low level of HBV-DNA (387 IU/mL). The liver function tests were standard, with ALT measured at 13.0 U/L (**Supplementary Figures S9C**). The extended follow-up showed HBV-DNA decreasing to 225 IU/mL and becoming undetectable (<1.00 × 10² IU/mL), with HBsAg >250 IU/mL, HBeAg 2.64 PEIU/mL, ALT 12.0–24.9 U/L, AST 23.0–28.0 U/L, and γ-GGT 24.0-31.4 U/L. Entecavir prophylaxis was maintained, with no HBV reactivation or liver dysfunction, demonstrating sustained viral control.

As of the date of submission, the patient’s condition remains stable with no significant changes in her clinical status.

## 3 Literature review

In recent years, the treatment paradigm for locally advanced rectal cancer (LARC) has evolved from a surgery-centric approach to a multidisciplinary model anchored by neoadjuvant chemoradiotherapy (nCRT). The adoption of nCRT has significantly enhanced local control by reducing tumor burden, increasing resectability, and improving R0 resection rates, thereby establishing itself as the standard of care for LARC. However, its limitations in improving long-term outcomes, including disease-free survival (DFS) and overall survival (OS), as well as controlling distant metastases remain evident. These shortcomings have driven efforts to refine treatment strategies. Total neoadjuvant therapy (TNT), which incorporates systemic chemotherapy alongside nCRT, has emerged as a promising approach to enhance tumor downstaging further and reduce the recurrence rates. Meanwhile, the advent of immunotherapy, particularly immune checkpoint inhibitors (ICIs), has introduced novel opportunities to optimize neoadjuvant strategies.

While nCRT has achieved substantial improvements in local control, its inherent limitations are well documented. The CAO/ARO/AIO-94 trial (13) marked a pivotal advance, demonstrating that preoperative nCRT significantly improved pathologic downstaging and sphincter preservation rates while reducing long-term local recurrence rates compared to postoperative chemoradiotherapy. However, no significant improvements in DFS or OS were observed. Similarly, the TROG 01.04 trial (14) compared neoadjuvant chemoradiotherapy (nCRT) with short-course radiotherapy (SCRT) in T3 rectal cancer, finding that while nCRT achieved superior pathologic complete response (pCR) and tumor downstaging rates, there were no significant differences between the two approaches in 5-year survival or long-term toxicity. The RECTAL-BOOST trial (15) investigated whether intensifying radiotherapy doses (15 Gy/3 fractions + 50 Gy/25 fractions with concurrent capecitabine) could enhance the pathological complete response (pCR) rates. Although tumor downstaging and near-complete response (near-pCR) rates improved (from 45.3% to 69.4%), no significant increase in pCR rates was observed, and higher toxicity was reported. In contrast, the PROSPECT trial (16) evaluated mFOLFOX6 neoadjuvant chemotherapy as an alternative to nCRT, showing comparable 5-year DFS and local recurrence rates, with significantly reduced toxicity in the chemotherapy group, particularly benefiting younger patients or those unable to tolerate radiotherapy. Collectively, these studies highlight the limitations of nCRT, including its modest pCR rates (15%–20%), limited efficacy in controlling distant metastases, and insufficient emphasis on preserving fertility and enhancing postoperative quality of life, particularly in younger patients.

To address these limitations, the integration of immunotherapy into neoadjuvant treatment has opened new avenues for LARC management. The VOLTAGE-A trial (17) was the first to combine ICIs with nCRT, demonstrating a pCR rate of 30% in MSS rectal cancer patients and an impressive 78% in MSI-H patients. Furthermore, the NSABP FR-2 (18) and PANDORA trials (19) explored the use of durvalumab (a PD-L1 inhibitor) as consolidation therapy following nCRT, with the PANDORA trial reporting a pCR rate of 32.7% and notable benefits in tumor downstaging and sphincter preservation. The NRG-GI002 trial (20) evaluated the addition of pembrolizumab within a TNT framework. While the pCR rates remained unchanged (29.4% vs. 31.9%), the 3-year OS significantly improved (87% vs. 95%), highlighting the potential long-term benefits of ICIs. In a Chinese study (PKUCH-04) (21), camrelizumab combined with nCRT achieved a pathological complete response (pCR) rate of 33% in patients with MSS rectal cancer, with no ≥4-grade adverse events, underscoring its safety and efficacy. Similarly, the TORCH trial (22) demonstrated the efficacy of SCRT combined with toripalimab and consolidation chemotherapy, achieving a pathological complete response (pCR) rate of 60.7% and an anal preservation rate of 88.9%. The interplay between chronic hepatitis B virus (HBV) infection and cancer immunotherapy represents an emerging area of interest, particularly in regions with high HBV prevalence such as Asia. Long-term antiviral therapy outcomes demonstrate that while HBV-DNA negativity can be achieved in 97.7% of patients treated with tenofovir or entecavir for at least 10 years, HBsAG clearance remains rare (2.9%), indicating persistent viral reservoir and ongoing hepatocarcinogenic risk (23). This persistent viral presence has profound implications for modulating the tumor microenvironment and enhancing immunotherapy responses. In hepatocellular carcinoma (HCC), HBV infection is known to induce an immunosuppressive tumor microenvironment (TME), characterized by T-cell exhaustion and reduced infiltration of effector immune cells, which can diminish the efficacy of ICIs (24)—for instance, Pan et al. (2024) reported that higher HBV viral loads were associated with poorer responses to PD-1 inhibitors in advanced HCC, likely due to virus-induced immune tolerance (24).

The molecular mechanisms underlying HBV’s impact on the tumor microenvironment are increasingly understood through the role of APOBEC3 family proteins. The APOBEC3 family of cytidine deaminases represents a crucial component of innate antiviral immunity against HBV, exerting anti-HBV effects through multiple mechanisms including G-to-A hypermutation of viral DNA, suppression of HBV gene transcription, and inhibition of viral particle formation (25). However, these proteins function as a double-edged sword—while providing antiviral protection, their aberrant expression and improper regulation can threaten genomic stability and contribute to hepatocarcinogenesis.

APOBEC3B specifically plays a pivotal role in HBV-related hepatocellular carcinoma progression through its dual functionality. While it can inhibit HBV replication by affecting the nuclear HBV DNA levels and reducing viral gene expression, it simultaneously induces C-to-T mutations in host cell genes, accelerating tumor progression (26). The tumor microenvironment in HBV-related HCC is significantly influenced by these virus–host interactions, with APOBEC3B contributing to the induction of HBx mutations that can overexpress PLA2R (phospholipase A2 receptor), activate the NLRP3 inflammasome in hepatocytes, and lead to the generation of reactive oxygen species (ROS) and accelerated inflammatory responses.

A critical positive feedback loop exists between APOBEC3B and IL-6 in the hepatic tumor microenvironment. APOBEC3B induces IL-6 overexpression by modulating human antigen R (HuR), enhancing IL-6 mRNA stability, and triggering persistent inflammatory reactions. Conversely, IL-6 upregulates APOBEC3B expression via the JAK1/STAT3 pathway, creating a pro-inflammatory microenvironment that facilitates HCC evolution. Furthermore, APOBEC3B promotes the upregulation of chemokine expression, leading to the recruitment of myeloid-derived suppressor cells (MDSCs) and tumor-associated macrophages (TAMs), which inhibit CD8+ T cell function and create an immunosuppressive microenvironment conducive to liver cancer development (26).

Conversely, some studies suggest that HBV-driven chronic inflammation may enhance TME immunogenicity in specific contexts. Zheng et al. (2024) identified distinct immunophenotypes in HBV-positive HCC, including an increased expression of pro-inflammatory cytokines such as IL-6 and TNF-α, which could potentially sensitize tumors to immunotherapy (27). Limited data exist on HBV’s impact on immunotherapy in non-hepatic malignancies such as colorectal cancer. Nuersulitan et al. (2022) found that HBV infection did not significantly alter outcomes in patients with diffuse large B-cell lymphoma (DLBCL) receiving chemotherapy; however, the risk of viral reactivation remained a critical concern (28). A study by Li et al. (2023) further highlighted that HBV-positive patients with HCC exhibited variable responses to PD-1/PD-L1 inhibitors, with some benefiting from enhanced inflammatory signaling within the TME (29). Emerging evidence from HCC studies suggests that chronic HBV infection upregulates PD-L1 expression in the TME, potentially increasing tumor susceptibility to PD-1/PD-L1 blockade due to altered immune signaling pathways.

Additionally, Xu (2025) found that the serum levels of cytokines such as CCL19 in HBV-positive HCC patients were predictive of better responses to anti-PD-1 therapy, indicating that HBV-induced inflammatory signals may enhance immune activation in some instances (30). Combining PD-1/PD-L1 inhibitors with tyrosine kinase inhibitors (TKIs) has been shown to stabilize the immune contexture in HBV-positive HCC, creating a more favorable TME for immunotherapy (31). However, high HBV DNA levels have been associated with immunotherapy resistance and an increased incidence of immune-related adverse events (irAEs) in HCC, highlighting the importance of antiviral therapies in optimizing treatment outcomes (32). These findings underscore the complex, context-dependent role of HBV in modulating cancer treatment responses, particularly for MSS tumors, which are typically less responsive to ICIs. The paucity of studies specifically addressing HBV in colorectal cancer highlights the need for further research to elucidate its impact on immunotherapy efficacy and to identify biomarkers that could guide patient selection in this unique population. These findings underscore the significant potential of immunotherapy to improve outcomes in LARC, particularly in MSI-H patients, where some may even avoid surgery through neoadjuvant immunotherapy.

Overall, while nCRT remains the standard treatment for LARC, its limitations have catalyzed the exploration of optimized approaches, including the broader implementation of TNT and the integration of immunotherapy. The role of HBV in shaping immunotherapy outcomes remains an underexplored frontier, particularly in non-hepatic cancers, such as LARC. Given the potential of HBV to modulate TME immunogenicity, upregulate PD-L1 expression, and influence ICI responses through inflammatory cytokines like CCL19, further studies are warranted to explore these mechanisms in colorectal cancer and to assess the role of antiviral therapies in optimizing treatment outcomes. Future research should prioritize identifying optimal combinations of ICIs, chemotherapy, and radiotherapy alongside developing precise biomarkers to guide patient selection, with the ultimate goal of maximizing long-term survival and quality of life.

## 4 Discussion

This study demonstrates the promising potential of cmFOLFOXIRI combined with camrelizumab as a neoadjuvant treatment strategy for locally advanced rectal cancer (LARC), particularly in patients with microsatellite-stable (MSS) tumors. The regimen achieved a high pathological complete response (pCR) rate with manageable toxicity. This approach not only provides an important treatment option for MSS patients but also underscores the critical role of multimodal therapy in enhancing tumor downstaging and improving long-term outcomes. While nCRT remains the standard of care for LARC, its limitations in controlling distant metastases and improving overall survival (OS) have driven the exploration of total neoadjuvant therapy (TNT) and the integration of immunotherapy into treatment strategies. Immunotherapy, particularly immune checkpoint inhibitors (ICIs), has introduced unprecedented opportunities by modulating the tumor microenvironment to overcome the limitations of traditional treatments in MSS patients. However, the application of these innovative therapies also poses unique challenges, particularly in special populations such as patients with HBV infection.

The impact of HBV infection on cancer treatment and its management has gained increasing attention. As a significant immunosuppressive factor, HBV infection not only elevates the risk of viral reactivation during cancer therapy but also influences the efficacy of immunotherapy and chemotherapy by altering the tumor microenvironment. Chronic HBV infection is a well-established risk factor for hepatocellular carcinoma (HCC) and has been shown to affect the therapeutic outcomes of other malignancies—for instance, studies have demonstrated that HBV-infected patients receiving ICIs may experience diminished therapeutic efficacy due to virus-induced modifications in the tumor microenvironment (24). HBV can regulate host immune responses, creating a more immunosuppressive tumor microenvironment that compromises the antitumor efficacy of ICIs (33). This phenomenon is particularly evident in HCC, where the response to ICIs is notably inferior compared to other solid tumors (27).

In addition to its effects on immunotherapy, HBV reactivation remains a critical concern for cancer patients undergoing immunosuppressive treatments such as chemotherapy and targeted therapies. The incidence of HBV reactivation among patients receiving these treatments can be as high as 14%–72%, with risk depending on the type of chemotherapy and the patient’s HBV serologic status (28). Reactivation can lead to severe hepatic complications, disrupt cancer treatment continuity, and even necessitate treatment discontinuation (34). However, prophylactic antiviral therapy, such as tenofovir, has been shown to significantly reduce the risk of HBV reactivation, further underscoring the importance of HBV screening and management in cancer patients (35).

In the context of non-hepatic malignancies, the impact of HBV infection on treatment outcomes varies. While some studies suggest that HBV infection may not significantly affect the outcomes of specific therapies, such as chimeric antigen receptor (CAR) T-cell therapy (36), the overall immunosuppressive state induced by HBV still poses challenges for achieving optimal therapeutic responses in various cancers (37)—for example, HBV-positive patients with diffuse large B-cell lymphoma (DLBCL) face higher risks of viral reactivation during treatment, complicating clinical management (38). Furthermore, HBV viral load and serologic markers have been correlated with treatment responses and prognostic outcomes across different malignancies. In patients with liver cancer, higher HBV DNA levels have been associated with poorer responses to PD-1 inhibitors, suggesting that viral burden may directly influence the efficacy of immunotherapy (39).

In this case, the patient’s HBV status was effectively managed with entecavir prophylaxis, preventing reactivation and potentially preserving the immunogenicity of the TME, which may have contributed to the observed pathological complete response (pCR). The slight increase in HBV-DNA to 387 IU/mL during follow-up, without liver dysfunction, highlights the importance of vigilant monitoring and antiviral therapy in supporting multimodal treatment. These findings suggest that HBV-positive MSS LARC patients may represent a unique subgroup that could benefit from combined chemotherapy and immunotherapy, provided that viral control is maintained. Effective HBV management was critical to this case’s success. Biweekly HBV-DNA monitoring during neoadjuvant chemotherapy and monthly testing during chemoradiotherapy confirmed sustained viral suppression (undetectable to 387 IU/mL). Transient liver enzyme elevations (ALT up to 61.0 U/L, AST up to 61.8 U/L) were observed, likely due to chemotherapy or cirrhosis, but resolved without clinical impact. Entecavir (0.5 mg daily) prevented reactivation, supporting treatment continuity. These findings suggest that robust antiviral therapy may enhance immunotherapy efficacy by preserving TME immunogenicity. However, challenges such as frequent monitoring and long-term adherence warrant consideration (40). However, the precise mechanisms by which HBV modulates TME and immunotherapy efficacy in non-hepatic malignancies remain speculative, necessitating further mechanistic and clinical studies (38).

In summary, HBV infection complicates cancer management by altering immune responses and increasing the risk of viral reactivation during antitumor therapy. In this study, the patient received prophylactic antiviral therapy with entecavir, which successfully prevented HBV reactivation and provided valuable insights into managing similar cases. Future research should focus on optimizing treatment strategies for LARC, particularly for patients with HBV infection, by exploring individualized approaches and developing reliable biomarkers to identify those most likely to benefit from immunotherapy.

## 5 Limitations

As a single-case report, this study has inherent limitations in terms of generalizability. The pCR achieved in this HBV-positive MSS LARC patient suggests the potential efficacy of cmFOLFOXIRI plus camrelizumab; however, the results may be influenced by individual factors, such as tumor biology or HBV status. The lack of long-term follow-up data limits the evaluation of disease-free survival (DFS) and overall survival (OS). Additionally, reliance on pelvic CT and endoscopic ultrasonography (EUS) due to MRI contraindication may have reduced the staging precision. Larger, prospective, multicenter studies are needed to validate this approach in HBV-positive LARC patients and to explore the role of HBV in TME modulation.

## 6 Conclusion

This case report highlights the promising potential of cmFOLFOXIRI combined with camrelizumab as a neoadjuvant therapy for HBV-positive MSS LARC, achieving a pathological complete response (pCR) with manageable toxicity. Effective HBV management, including entecavir prophylaxis, was crucial in preventing reactivation and supporting treatment success. These preliminary findings underscore the need for larger studies to confirm the efficacy of this regimen, elucidate the immunomodulatory effects of HBV, and identify biomarkers to optimize treatment for this unique patient subgroup.

## Data Availability

The datasets generated and analyzed during this case report are publicly available on Zenodo and can be accessed via the following link: https://doi.org/10.5281/zenodo.14642563 (Li & Xu, 2025). The repository includes de-identified patient imaging, clinical data, and **Supplementary Materials** referenced in this manuscript. All data have been made available under a Creative Commons license, ensuring transparency and facilitating further research while safeguarding patient confidentiality.

## Supplementary material

Supplementary Figure 1 Colonoscopy image demonstrating a circumferential rectal mass. Colonoscopy reveals a circumferential mass in the rectum extending from the mid to lower segments (5 to 10 cm from the anal verge). The lesion measures approximately 5.5 cm in diameter and is characterized by an irregular surface with marked congestion and active bleeding, resulting in significant narrowing of the intestinal lumen.

Supplementary Figure 2 Immunohistochemical analysis of rectal adenocarcinoma.

Supplementary Figure 3 Endoscopic ultrasonography findings of rectal adenocarcinoma and suspicious lymph nodes. **(A)** Endoscopic ultrasonography (EUS) showing a hypoechoic rectal tumor involving the entire bowel wall thickness and reaching the serosal layer. The lesion is indicated by the red arrow and demonstrates irregular margins suggestive of malignant invasion. **(B)** EUS image highlighting suspicious perirectal lymph nodes (indicated by the red arrow) measuring 8.1 mm in diameter, suggesting possible nodal metastases.

Supplementary Figure 4 Pelvic CT imaging of rectal adenocarcinoma and associated findings. **(A)** Sagittal CT scan showing a thickened rectal wall (indicated by the red arrow) consistent with an irregular rectal mass invading the adjacent rectal layers. **(B)** Axial CT scan demonstrating the irregular thickening of the rectal wall (red arrow) and the resulting narrowing of the rectal lumen, suggesting advanced local tumor invasion. **(C)** Axial CT scan showing suspicious perirectal lymph nodes (red arrow), measuring >8 mm in short-axis diameter, indicating possible nodal metastases.

Supplementary Figure 5 Chest and abdominal CT imaging findings. **(A)** Chest CT scan showing mild chronic inflammation in the right lower lobe (indicated by the red arrows). No evidence of distant metastases is observed. **(B)** Abdominal CT scan demonstrating signs of cirrhosis (red arrow) with slightly irregular liver morphology. No definitive evidence of hepatic metastases is observed.

Supplementary Figure 6 Whole-body bone scintigraphy (ECT) findings. Whole-body bone scintigraphy (ECT) showing no abnormal tracer uptake, suggesting the absence of bone metastases.

Supplementary Figure 7 Follow-up CT images showing tumor regression and surrounding tissue changes after neoadjuvant chemotherapy. **(A)** Axial CT image showing reduced rectal wall thickness (red arrow) from 25 to 17 mm after neoadjuvant chemotherapy. Tumor involvement of the serosal layer has decreased. **(B)** Axial CT image showing the improved clarity of perirectal fat planes (red arrow) with stable perirectal lymph nodes.

Supplementary Figure 8 Histopathological examination of the rectal tumor bed post-treatment. **(A)** Hematoxylin and eosin (H&E) staining of the rectal tumor bed showing fibrous stromal hyperplasia and the absence of residual tumor cells, consistent with a complete pathological response (pCR). **(B)** Hematoxylin and eosin (H&E) staining showing the infiltration of histiocytes and fibrotic changes in the tumor bed, reflecting post-treatment remodeling and absence of malignancy.

Supplementary Figure 9 Follow-up imaging demonstrating absence of recurrence or metastasis. **(A)** Axial chest CT image showing no evidence of pulmonary metastases or abnormalities in the lung fields during follow-up. **(B)** Axial pelvic CT image highlighting the rectal anastomosis site (red arrow), demonstrating normal postoperative appearance with no signs of recurrence. **(C)** Sagittal pelvic CT image focusing on the rectal tumor bed (red arrow), revealing no residual disease or abnormal tissue proliferation during follow-up.
