# Supplementary figures and images for: Combined neoadjuvant chemotherapy and immunotherapy in a hepatitis B virus-positive patient with locally advanced rectal adenocarcinoma: a case report and literature review
Source: Front Oncol. 2025 Aug 20;15:1560508. doi: 10.3389/fonc.2025.1560508 (PMC12404937; doi:10.3389/fonc.2025.1560508)

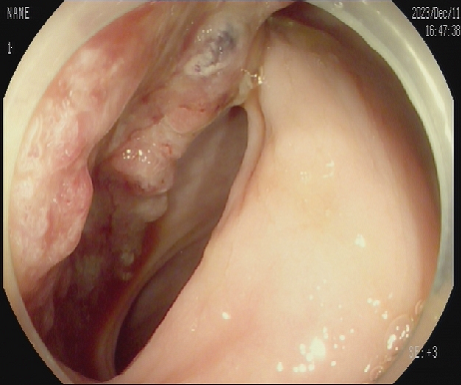

Supplement: Supplementary Figure 1 — Colonoscopy image demonstrating a circumferential rectal mass. Colonoscopy reveals a circumferential mass in the rectum extending from the mid to lower segments (5 to 10 cm from the anal verge). The lesion measures approximately 5.5 cm in diameter and is characterized by an irregular surface with marked congestion and active bleeding, resulting in significant narrowing of the intestinal lumen. [file Image1.tif]

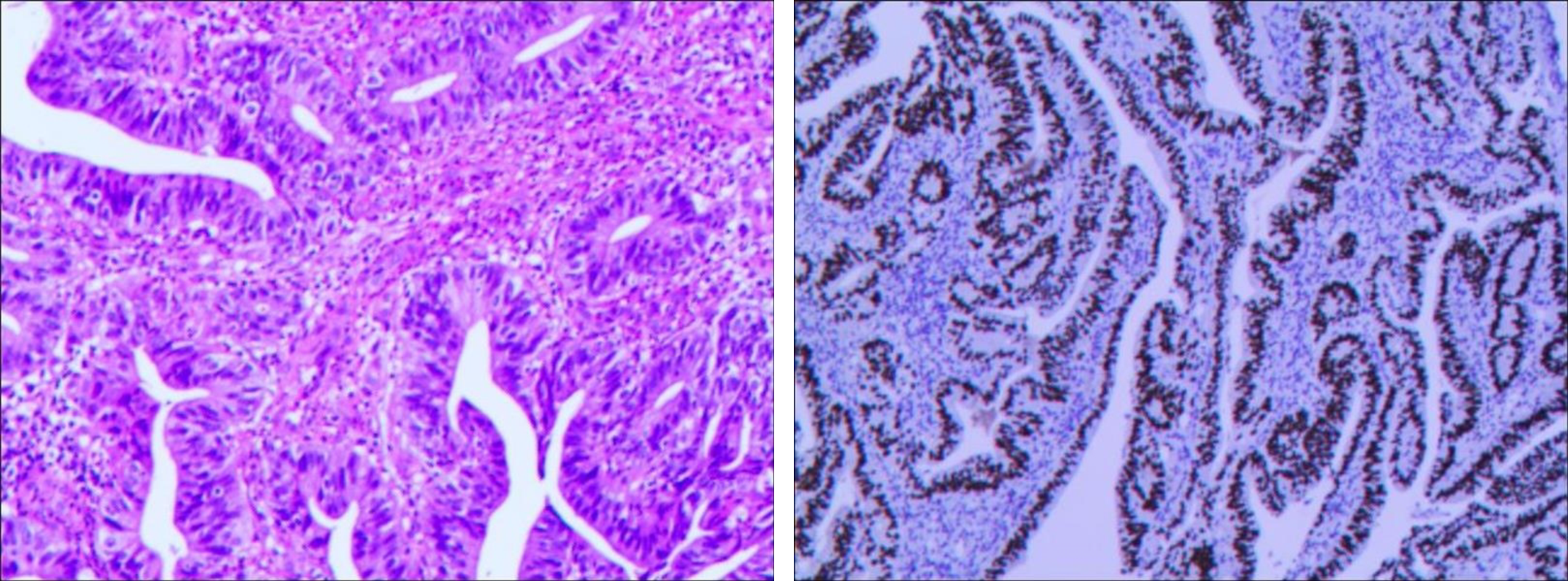

Supplement: Supplementary Figure 2 — Immunohistochemical analysis of rectal adenocarcinoma. [file Image2.tif]

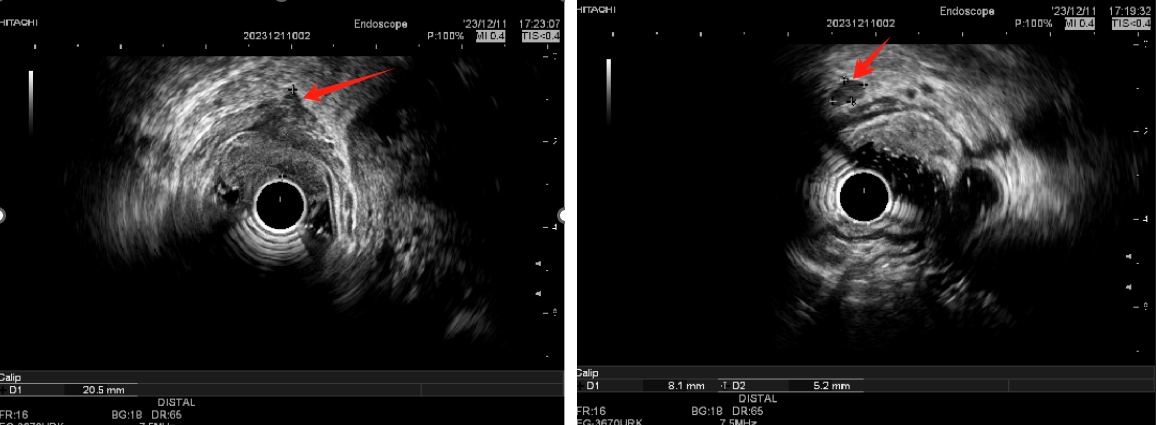

Supplement: Supplementary Figure 3 — Endoscopic ultrasonography findings of rectal adenocarcinoma and suspicious lymph nodes. (A) Endoscopic ultrasonography (EUS) showing a hypoechoic rectal tumor involving the entire bowel wall thickness and reaching the serosal layer. The lesion is indicated by the red arrow and demonstrates irregular margins suggestive of malignant invasion. (B) EUS image highlighting suspicious perirectal lymph nodes (indicated by the red arrow) measuring 8.1 mm in diameter, suggesting possible nodal metastases. [file Image3.tif]

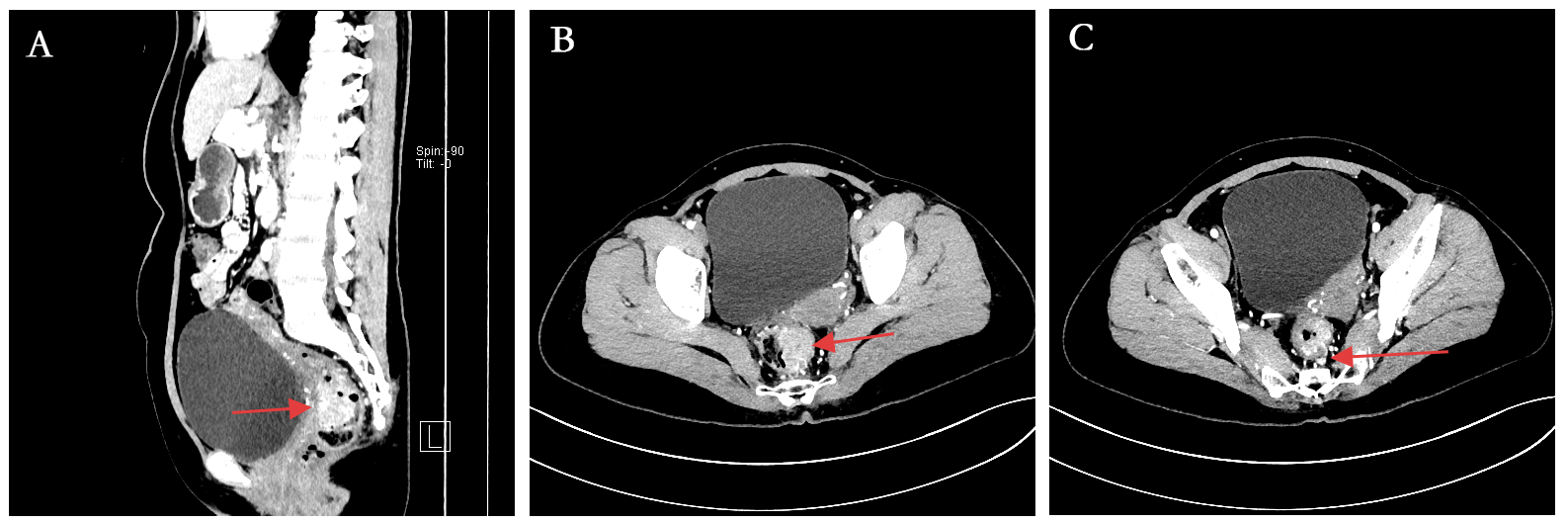

Supplement: Supplementary Figure 4 — Pelvic CT imaging of rectal adenocarcinoma and associated findings. (A) Sagittal CT scan showing a thickened rectal wall (indicated by the red arrow) consistent with an irregular rectal mass invading the adjacent rectal layers. (B) Axial CT scan demonstrating the irregular thickening of the rectal wall (red arrow) and the resulting narrowing of the rectal lumen, suggesting advanced local tumor invasion. (C) Axial CT scan showing suspicious perirectal lymph nodes (red arrow), measuring >8 mm in short-axis diameter, indicating possible nodal metastases. [file Image4.tif]

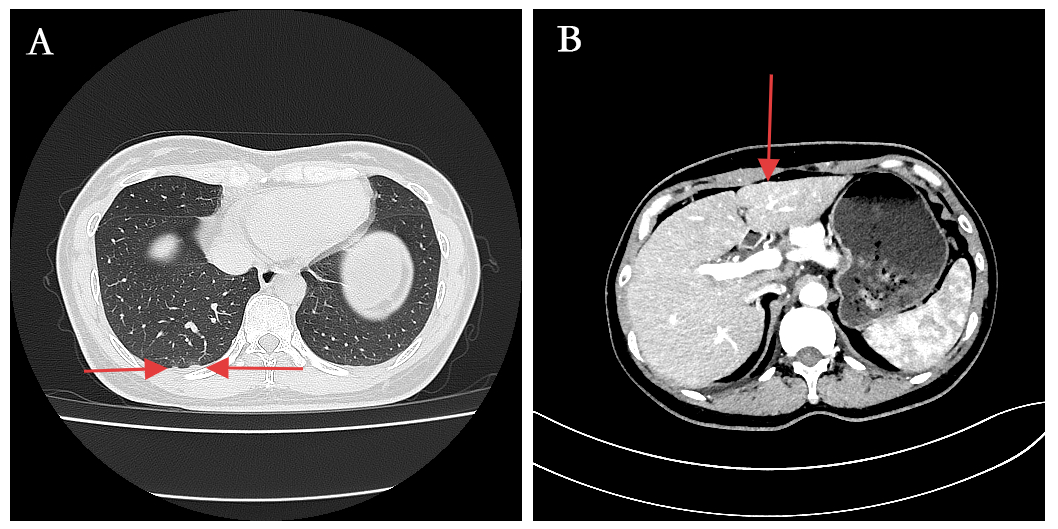

Supplement: Supplementary Figure 5 — Chest and abdominal CT imaging findings. (A) Chest CT scan showing mild chronic inflammation in the right lower lobe (indicated by the red arrows). No evidence of distant metastases is observed. (B) Abdominal CT scan demonstrating signs of cirrhosis (red arrow) with slightly irregular liver morphology. No definitive evidence of hepatic metastases is observed. [file Image5.tif]

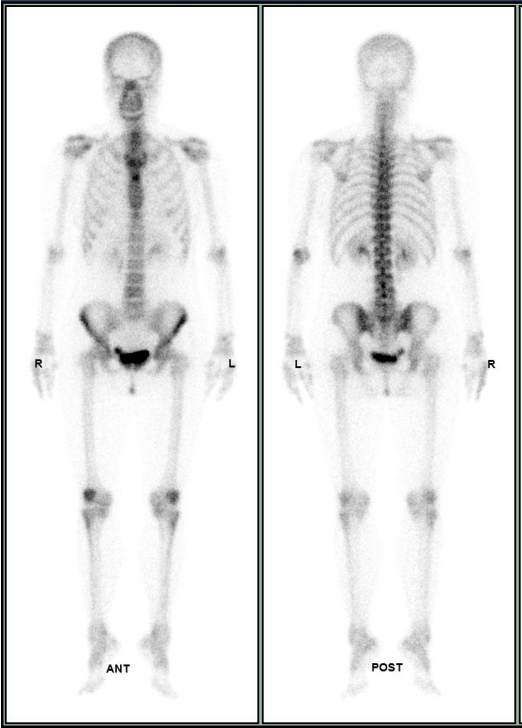

Supplement: Supplementary Figure 6 — Whole-body bone scintigraphy (ECT) findings. Whole-body bone scintigraphy (ECT) showing no abnormal tracer uptake, suggesting the absence of bone metastases. [file Image6.tiff]

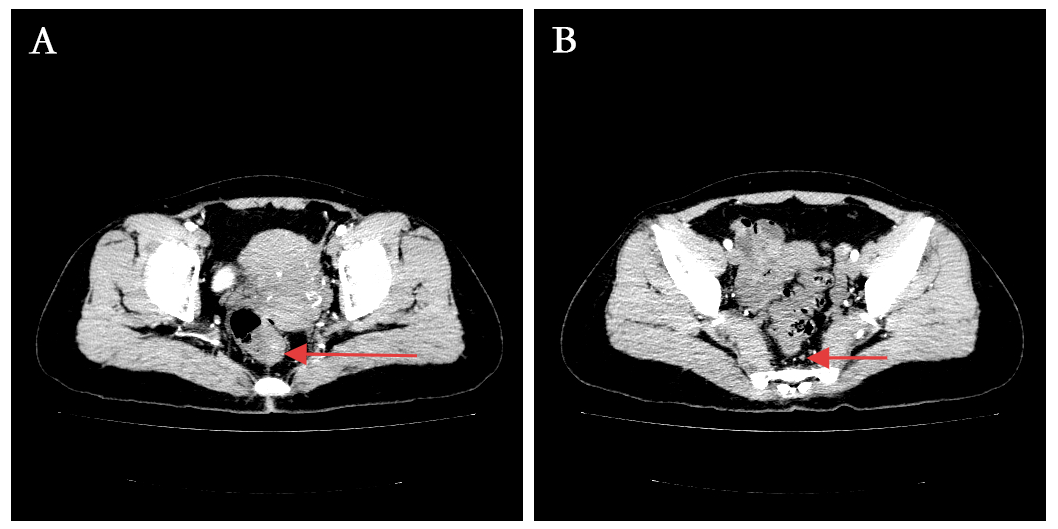

Supplement: Supplementary Figure 7 — Follow-up CT images showing tumor regression and surrounding tissue changes after neoadjuvant chemotherapy. (A) Axial CT image showing reduced rectal wall thickness (red arrow) from 25 to 17 mm after neoadjuvant chemotherapy. Tumor involvement of the serosal layer has decreased. (B) Axial CT image showing the improved clarity of perirectal fat planes (red arrow) with stable perirectal lymph nodes. [file Image7.tif]

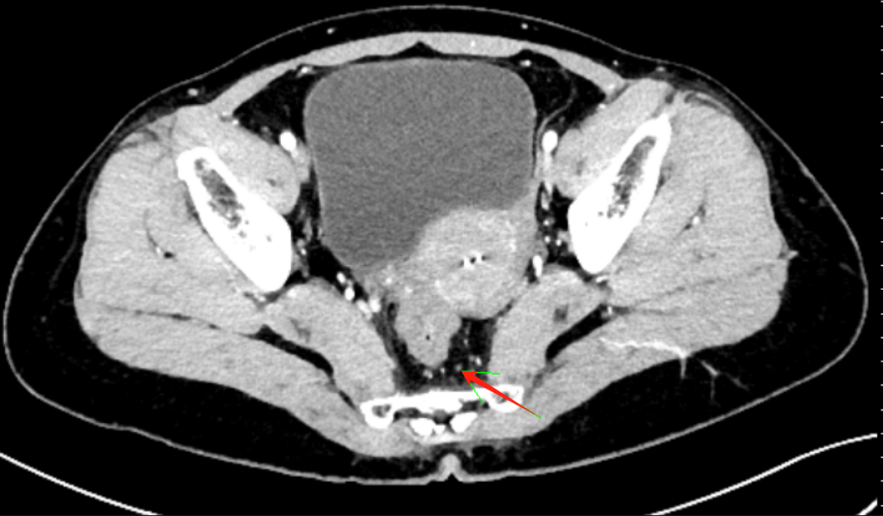

Supplement: Supplementary Figure 8 — Histopathological examination of the rectal tumor bed post-treatment. (A) Hematoxylin and eosin (H&E) staining of the rectal tumor bed showing fibrous stromal hyperplasia and the absence of residual tumor cells, consistent with a complete pathological response (pCR). (B) Hematoxylin and eosin (H&E) staining showing the infiltration of histiocytes and fibrotic changes in the tumor bed, reflecting post-treatment remodeling and absence of malignancy. [file Image8.tif]

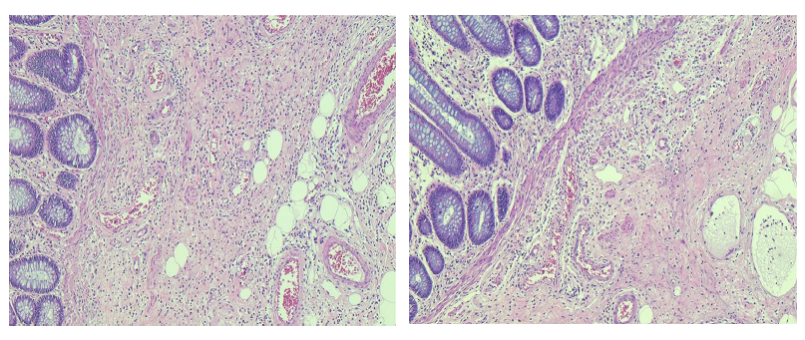

Supplement: Supplementary Figure 9 — Follow-up imaging demonstrating absence of recurrence or metastasis. (A) Axial chest CT image showing no evidence of pulmonary metastases or abnormalities in the lung fields during follow-up. (B) Axial pelvic CT image highlighting the rectal anastomosis site (red arrow), demonstrating normal postoperative appearance with no signs of recurrence. (C) Sagittal pelvic CT image focusing on the rectal tumor bed (red arrow), revealing no residual disease or abnormal tissue proliferation during follow-up. [file Image9.tif]

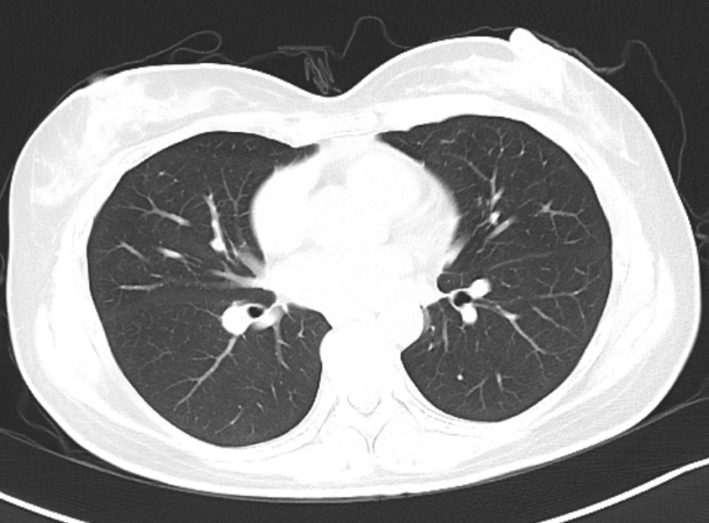

Supplement: Supplementary file 10 [file Image10.tif]

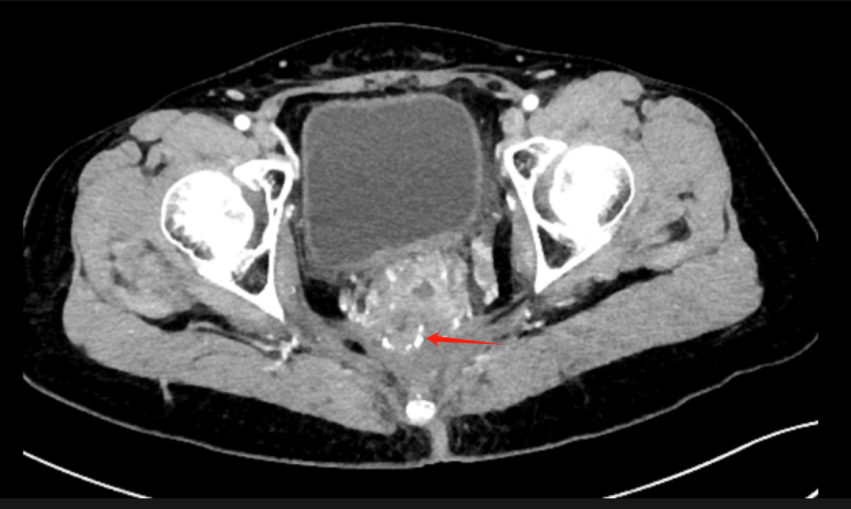

Supplement: Supplementary file 11 [file Image11.tif]

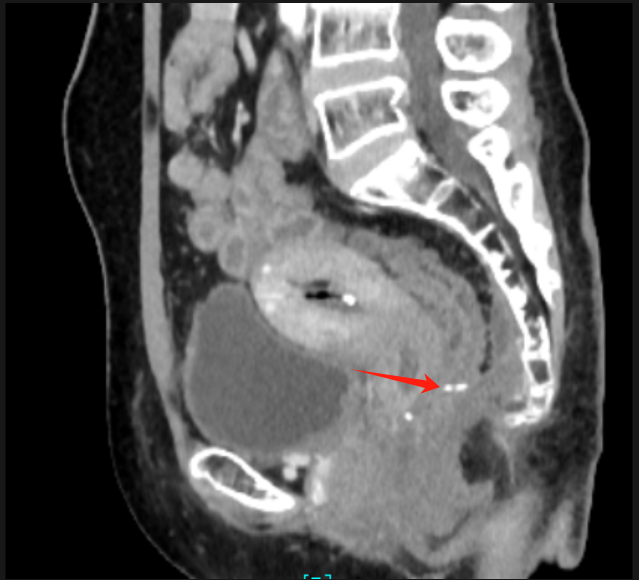

Supplement: Supplementary file 12 [file Image12.tif]
